# Clinical efficacy evaluation of pedicled vascularized iliac bone graft transfer for osteonecrosis of the femoral head: a retrospective study with DCE-MRI/CTP analysis

**DOI:** 10.3389/fsurg.2025.1696430

**Published:** 2025-11-21

**Authors:** Tianchen Zhang, Shanbin Zheng, Yuyang Zhai, Xinyi Hou, Yawen Wang, Zhiyuan Chen, Xun Cao, Zilei Xia, Tianwei Xia, Jirong Shen

**Affiliations:** 1 Affiliated Hospital of Nanjing University of Chinese Medicine, Nanjing, Jiangsu, China; 2 Nanjing University of Chinese Medicine, Nanjing, Jiangsu, China

**Keywords:** osteonecrosis of the femoral head, pedicled vascularized iliac bone graft transfer, hip preservation surgery, dynamic contrast-enhanced MRI, CT perfusion

## Abstract

**Objective:**

To evaluate the clinical efficacy of pedicled vascularized iliac bone graft transfer (PVIBGT) in treating osteonecrosis of the femoral head (ONFH) at Association Research Circulation Osseous (ARCO) stages II–IIIB, to assess postoperative revascularization using standardized dynamic contrast-enhanced magnetic resonance imaging (DCE-MRI) and CT perfusion imaging (CTP), and to identify independent predictors of surgical success through multivariate analysis.

**Methods:**

A retrospective analysis was conducted on 50 patients (51 hips) with ARCO stage II–IIIB ONFH (aged 18–48 years) who underwent PVIBGT between March 2021 and November 2024. Functional outcomes were assessed using Harris score. Radiographic evaluations (x-ray, CT) were used to assess bone graft integration and femoral head morphology. DCE-MRI parameters (IAUGC, CER, MaxSlope, Ktrans, Kep, Ve) and postoperative CTP indices (BF, BV, MTT) were used to quantify perfusion and microcirculatory status. Multivariate binary logistic regression including ARCO stage, etiology, age, sex, disease duration, and graft dimensions (length, diameter) was performed to identify independent predictors of hip-preservation success. Analyses were conducted using SPSS 26.0.

**Results:**

The mean follow-up duration was 15.1 months. Harris score improved significantly from a preoperative value of 62.39 ± 6.73 (median = 64.00, interquartile range = 10.00) to a postoperative value of 81.88 ± 10.20 (median = 85.00, interquartile range = 9.00) (*P* < 0.001). The clinical success rate was 80.39% (41/51 hips), and the radiographic success rate was 70.59% (36/51 hips). Logistic regression revealed no independent predictors among etiology, ARCO stage, sex, age, disease duration, or graft dimensions (*P* > 0.05). DCE-MRI showed partial normalization of perfusion in the femoral head, while CTP indicated favorable hemodynamic status in the operated hip. Ten hips (19.61%) required THA due to femoral head collapse (>2 mm, 6 cases), joint space narrowing with persistent pain (3 cases), or poor function (1 case).

**Conclusion:**

PVIBGT effectively improves hip function and delays femoral head collapse in ARCO II–IIIB ONFH. Combined DCE-MRI and CTP provide objective imaging evidence for postoperative revascularization assessment. No single factor independently predicted success, possibly due to limited sample size and subgroup heterogeneity. Larger, prospective multicenter studies with standardized imaging protocols and extended follow-up are warranted to confirm these findings.

## 1 Introduction

Osteonecrosis of the femoral head (ONFH) is a common joint disease in orthopedics, which is prevalent in young and middle-aged people (1–3), and if not intervened in a timely manner, about 70% of the patients will suffer from femoral head collapse within a few years, which can lead to severe disability to the point where they face total hip arthroplasty (THA). If THA is accepted too early, its potential complications and revisions will be inevitable. Therefore, hip-preserving surgery has more advantages for young and middle-aged people compared to THA (4). However, there are currently numerous approaches of hip-preserving surgery, and how to choose the hip-preserving plan and the clinical efficacy of different strategies are still worth discussing (5–7). According to existing literature reports (8–13), the main hip-preserving surgical methods include core decompression, intertrochanteric rotational osteotomy, fibular support technique, porous tantalum metal rod implantation, etc. However, the above-mentioned surgical methods have objective curative effects for ONFH patients in Association Research Circulation Osseous (ARCO) stage I and II, but the clinical prognosis for ONFH in ARCO stage III is not good (14). The Pedicled Vascularized Iliac Bone Graft Transfer (PVIBGT) is an important surgical method for the treatment of early and middle-stage osteonecrosis of the femoral head, and is suitable for patients with ONFH in ARCO stage II–IIIB or Ficat stage II–IV (15–17). This surgical technique can restore mechanical support to the osteonecrotic region of the femoral head, alleviate symptoms, enhance local blood circulation, successfully impede the advancement of the necrotic area, and delay or even avert total hip arthroplasty (18–20). Patients aged over 50 with extensive femoral head collapse, cartilage surface rupture, or defect in ARCO stage IIIC–IV are frequently deemed unsuitable for this surgical approach. Conversely, pertinent studies indicate that young ONFH patients experience a relatively significant therapeutic effect from this surgical method, irrespective of the presence of extensive femoral head collapse (21, 22).

Certain studies indicate that while PVIBGT can restore blood supply to the femoral head and postpone the collapse of the necrotic region, issues such as bone graft absorption, insufficient mechanical support and complications in the donor area may occur after surgery, leading to the failure of hip preservation. Therefore, its therapeutic efficacy and influencing factors still need further study (6, 7, 23). Presently, there is an abundance of research about the treatment of ONFH using PVIBGT; nevertheless, an evaluation of efficacy utilizing dynamic contrast-enhanced MRI (DCE-MRI) and computed tomography perfusion imaging (CTP) remains lacking. For this reason, this study retrospectively analyzed 50 cases (51 hips) of ONFH patients treated with PVIBGT from March 2021 to November 2024, and combined DCE-MRI and CTP for efficacy evaluation. The findings are presented as follows.

## 2 Materials and methods

### 2.1 Inclusion and exclusion criteria for cases

#### 2.1.1 Inclusion criteria

(1) Patients aged 18–50 years old; (2) Patients with ARCO stage II to IIIB ONFH according to the ARCO staging criteria (24, 25); (3) All were treated with PVIBGT; (4) Patients with a strong willingness to preserve the hip and agreed to complete the entire study process as required by the protocol; (5) Patients understood the purpose of the trial and voluntarily signed the informed consent form.

#### 2.1.2 Criteria for exclusion

(1) Patients with severe hip osteoarthritis before surgery; (2) Patients with suspected infections; (3) Patients experiencing acute cardiovascular or cerebrovascular conditions, coagulation disorders, significant trauma, or major surgical procedures, including hip surgery on the affected side, within 6 months preceding the operation; (4) Patients with malignancies, a documented history of mental health disorders, or previous infectious diseases; (5) Pregnant and lactating patients; (6) Patients in the active stage of rheumatic immune diseases before surgery and requiring large doses of hormones.

### 2.2 General information

A total of 50 cases (51 hips) were analysed in this study. The age of participants ranged from 18 to 48 years, with a mean age of 31.88 ± 7.42 years. Among the cases, 37 were male (38 hips) and 13 were female (13 hips). The distribution of hips was 26 on the left side and 25 on the right side. The aetiology of ONFH included 18 cases (19 hips) classified as steroid-induced, 12 cases (12 hips) as alcohol-induced, 17 cases (17 hips) as idiopathic, and 3 cases (3 hips) as traumatic. The disease duration ranged from 0.5 to 25 months, with a mean of 6.81 ± 6.65 months. The length of hospital stay varied between 6 and 44 days, averaging 15.92 ± 6.10 days. The duration of the operation spanned from 70 to 405 min, with an average of 191.96 ± 65.77 min. See Table 1 for details.

**Table 1:** General information.

Characteristics	Value/Number of cases (%)
Total patients (hips)	50 (51)
Age (years)	Range: 18–48; mean: 31.88 ± 7.42
Gender	Male: 37 (38 hips); female: 13 (13 hips)
Affected side	Left: 26 hips; right: 25 hips
Etiology	Hormonal: 19 hips; alcoholic: 12 hips; idiopathic: 17 hips; traumatic: 3 hips
Disease duration	Range: 0.5–25 months; mean: 6.81 ± 6.65 months
Hospital stay	Range: 6–44 days; mean: 15.92 ± 6.10 days
Operative time	Range: 70–405 min; mean: 191.96 ± 65.77 min

### 2.3 Surgical technique

**Figure 1:**
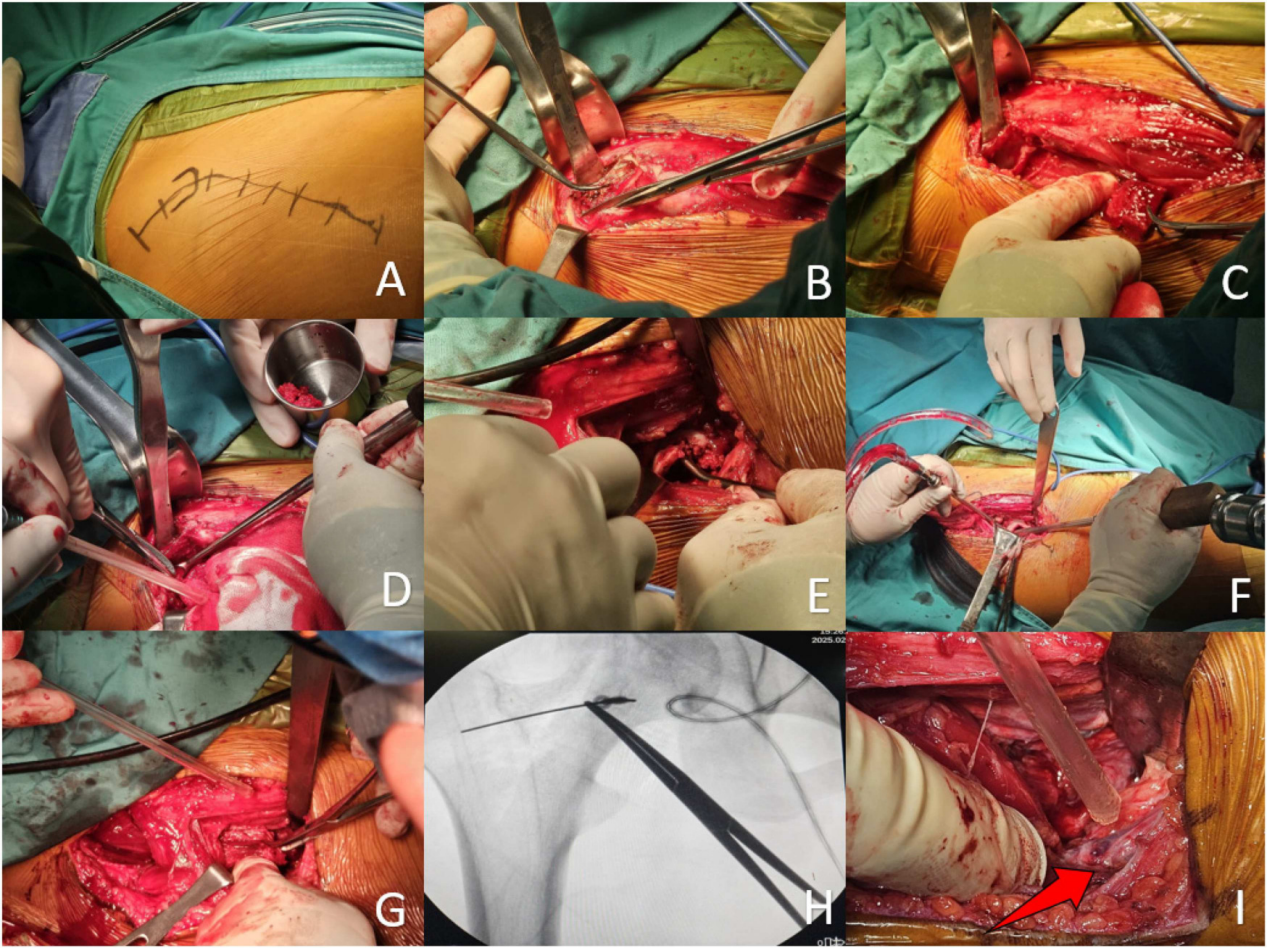
Surgical steps of the transfer of the iliac flap utilizing the ascending branch of the lateral femoral circumflex artery (ALFCA). **(A)** Marking of the Smith-Petersen (SP) incision; **(B)** Taking the outer plate of the ilium, retaining the muscle pedicle and cancellous bone; **(C)** Freeing the iliac flap (the iliac flap shows rich blood supply in Figure **C**); **(D)** Obtaining cancellous bone simultaneously; **(E)** Sclerotic bone in the necrotic region of the femoral head is removed using a high-speed drill and curette; **(F)** Filling and reinforcing the necrotic area with cancellous bone; **(G)** Dissecting the vessels of the ALFCA; **(H)** Implanting and fixing the iliac flap with the blood supply of the ALFCA (the cancellous bone surface facing the femoral head) through the bone window to the anterolateral column; **(I)** The ALFCA seen during the operation (the red arrow indicates the course of the vessel).

**Figure 2:**
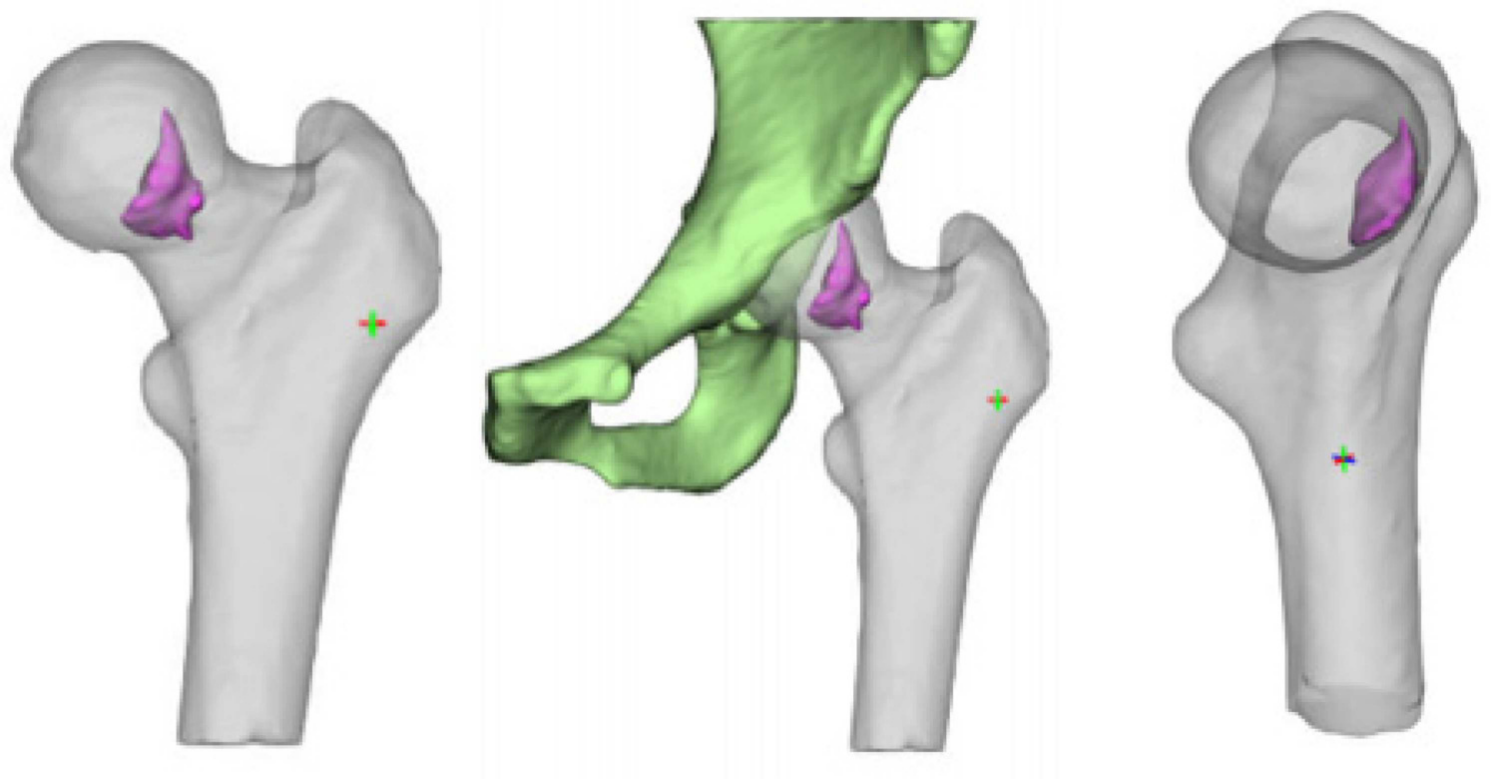
Schematic diagram of iliac flap implantation.

All surgeries were performed by one senior doctor. The surgical procedure is detailed as follows: Following general anaesthesia, the patient was positioned supine, with the affected hip elevated at a 30° angle. An incision was created along the trajectory from the anterior superior iliac spine to the lateral border of the patella, extending from the iliac crest to 3 cm proximal to the anterior inferior iliac spine, and laterally towards the greater trochanter, resulting in an 8–12 cm incision. When dissecting the skin and subcutaneous tissues layer by layer, it is essential to protect the lateral femoral cutaneous nerve and employ the double retractor technique. This involves exposing the sartorius-rectus femoris complex medially and stretching the tensor fascia lata laterally to fully reveal the Huter space. Dissect the ascending branch of the lateral femoral circumflex artery (ALFCA) at the distal end of the space, as this vessel supplies blood to the iliac bone graft. Trace its branches from the origin to the proximal end to separate the tensor fascia lata at the insertion point on the anterior superior iliac spine. Prepare a cubic iliac bone block originating from the tensor fascia lata while simultaneously obtaining cancellous bone. After incising the hip joint capsule to expose the femoral head, excise the subchondral necrotic bone and meticulously debride the sclerotic bone using a high-speed burr and curette. Fill the necrotic region with cancellous bone and apply tamping for reinforcement. Position the cancellous bone surface of the vascularized myocutaneous bone graft against the inner aspect of the femoral head. Transfer the iliac bone graft via the bone window to reinforce the anterolateral column, and secure it with screws. After securing the bone graft, irrigate with normal saline and proceed to close the incision in layers. It is essential to safeguard the lateral femoral cutaneous nerve during the procedure. See Figures 1, 2.

## 3 Postoperative management and postoperative follow-up

The operation duration and hospital stay of each case were completely recorded. The patients were immediately instructed to perform isometric contraction training of the lower extremity muscle groups after the operation. Weight-bearing was gradually started with the assistance of a walking aid 3 months after the operation, and a complete weight-bearing state was gradually transitioned to 6 months later.

### 3.1 Follow-up protocol

**Figure 3:**
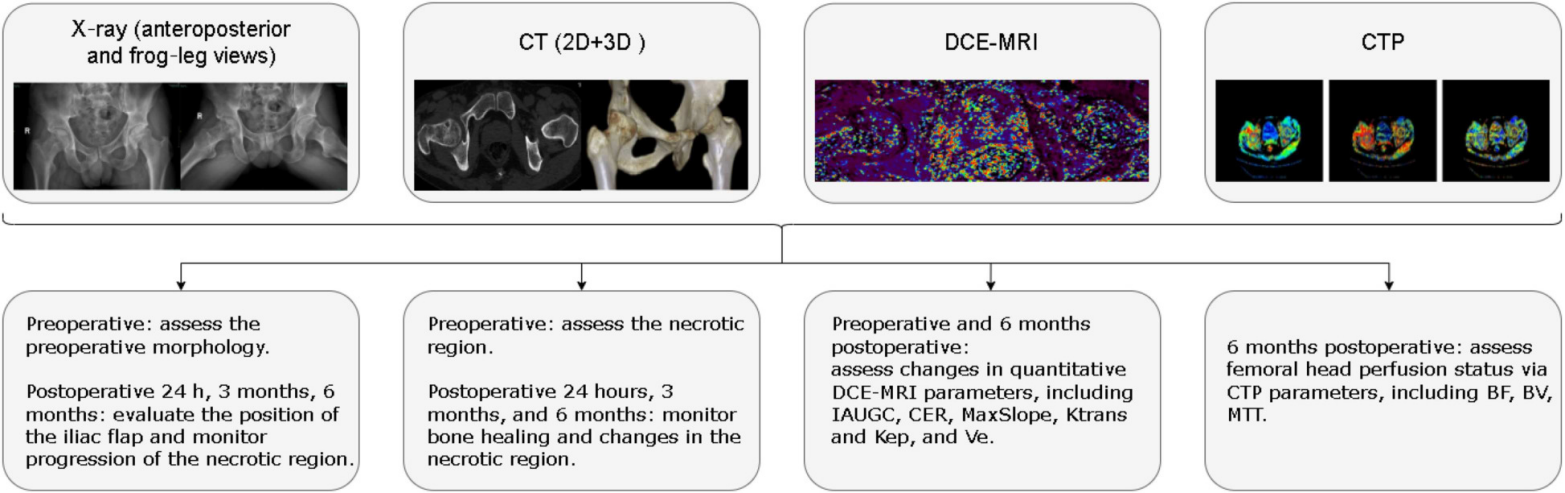
Imaging evaluation protocol for PVIBGT in the treatment of ONFH.

1. Harris Scoring System: The Harris Scoring System was employed to evaluate functional recovery post-surgery, with excellent and good rates (≥80 points) determined from the final follow-up data. The evaluation criteria were: Excellent (90–100), Good (80–89), Fair (70–79), and Poor (≤69).
2. Imaging Evaluation: Imaging evaluation was performed as described below (Figure 3).
3. X-ray examination: Anteroposterior and frog-leg views were taken before the operation, 24 h after the operation, 3 and 6 months after the operation to assess the morphological alterations of the necrotic region and the general status of the femoral head.
4. CT scan: CT scans were conducted concurrently with the x-ray examination to assess the necrotic region of the femoral head, the placement of the iliac flap, and the integration of the bone.
5. DCE-MRI: Examinations were performed before and 6 months after the operation. The scans were completed on a 3.0T MRI scanner (SIGNA Architect, GE Healthcare, USA) using a DISCO sequence (TR/TE = 4.2/1.5 ms, flip angle 12°, slice thickness 1 mm, matrix 360 × 360, FOV 36 cm × 36 cm), with a temporal resolution of 12.7 s/phase for a total of 15 phases. The contrast agent used was gadoterate meglumine (0.4 mL/kg, injection rate 3 mL/s), and dynamic scanning commenced 10 s after injection. Data were processed using GE Geniq software, with Regions of Interest (ROIs) set in the necrotic area and the edema area, covering an area range of 12.8–95.7 mm^2^ (mean 43.71 ± 15.50 mm^2^). Quantitative parameters include the initial area under the gadolinium concentration–time curve (IAUGC, indicating total blood flow), contrast enhancement ratio (CER, reflecting capillary density), maximum slope of enhancement (MaxSlope, representing blood flow velocity), volume transfer constant (Ktrans) and rate constant (Kep, both indicative of transcapillary exchange capacity), and extravascular extracellular volume fraction (Ve, reflecting tissue edema), to comprehensively evaluate the microcirculation status of the femoral head before and after the operation.
6. CTP examination: CT perfusion imaging was performed 6 months after the operation to evaluate the blood supply to the femoral head. The examination was conducted using a 256-slice CT scanner (Revolution, GE Healthcare, USA) with the following parameters: 80 kV, 80 mA, slice thickness 5 mm, matrix 512 × 512, FOV 40 cm. Ioversol (350 mgI/mL, 50 mL, 4 mL/s) was injected, and scanning was initiated after a 10-second delay, acquiring 20 volumetric datasets over a total duration of 65 s. Images were processed on a GE AW 4.7 workstation, with the femoral artery used as the input artery to generate CT perfusion images. Spherical ROIs were placed over the entire femoral head region on bone window axial images. The software automatically calculated parameters such as blood flow (BF), blood volume (BV), and mean transit time (MTT) to quantify the perfusion status of bone tissue and assist in judging the effect of revascularization after the operation.

The DCE-MRI and CTP data presented in this study were obtained from representative postoperative cases to illustrate the perfusion characteristics of the femoral head and to demonstrate the visualization and quantitative analysis workflow. These examples are intended to show the methodological application of DCE-MRI and CTP rather than to provide statistical comparisons for the entire cohort. Due to the exploratory nature of the present study and the incomplete availability of longitudinal imaging data for all patients, full-cohort quantitative analysis was not included at this stage.

7. Monitoring of Complications: Throughout the follow-up period, the incision healing was observed, and infection was screened in combination with blood routine and C-reactive protein (CRP) tests; deep vein thrombosis was screened by lower extremity venous ultrasound.

### 3.2 Evaluation indicators of curative effect

This study comprehensively evaluated the clinical efficacy and revascularization outcome of PVIBGT for ONFH using multi-dimensional indices, which primarily include: (1) Surgical success rate; (2) Assessment of imaging conditions at the vascularised iliac bone graft implantation site at 3 and 6 months post-surgery; (3) Harris score comparison before and 6 months post-surgery; (4) Incidence of continued femoral head collapse at 6 months post-surgery; (5) CT observation at 6 months post-surgery; (6) Changes in femoral head microcirculation before and 6 months post-surgery assessed via DCE-MRI; (7) The hemodynamics of the femoral head after the operation was observed by CTP to assist in judging the effect of revascularization. Specifically, the definition of clinical success refers to the research criteria of Mont et al. (26), Zhang et al. (27), and Li et al. (28). The specific criteria are as follows: Harris score ≥ 80, no further progression of femoral head collapse, and no need for total hip arthroplasty during the follow-up period. Radiological success is defined as no progression of necrotic collapse or joint space narrowing, with maintenance of a continuous Shenton's line on radiographs.

### 3.3 Statistical techniques

Statistical analyses were conducted utilising Statistical Package for the Social Sciences (SPSS) version 26.0 (IBM Corp., USA). Measurement data are presented as mean ± standard deviation (*x¯* ± SD). Normality of data distribution was assessed using the Shapiro–Wilk test. Paired *t*-tests were employed for within-group comparisons of normally distributed data. The Wilcoxon signed-rank test was employed to analyse non-normally distributed data. The significance threshold (*α*) was established at 0.05, with a *P*-value below this threshold deemed statistically significant.

### 3.4 Multivariate logistic regression analysis

To further explore the independent predictive factors for the success or failure of PVIBGT, this study used the multivariate binary Logistic regression method to analyze variables such as ARCO stage, etiology, age, gender, disease duration, maximum length and diameter of the bone graft. Two models were established: Model 1 (Block 1) included the etiology types (hormonal, alcoholic, idiopathic, traumatic) and ARCO stage to control disease-related confounding factors; Model 2 (Block 2) included gender, age, disease duration, length and maximum diameter of the iliac bone graft to evaluate the impact of the patient's basic situation and surgery-related factors. All variables were entered into the model at once using the “Enter method”. The dependent variable was the surgical outcome (success = 1, failure = 0). The odds ratio (OR) of each variable and its 95% confidence interval (CI) were calculated. The goodness of fit of the model was evaluated by the Hosmer–Lemeshow test. A *P* > 0.05 indicated a good fit.

## 4 Results

### 4.1 Harris score and clinical and imaging results

**Figure 4:**
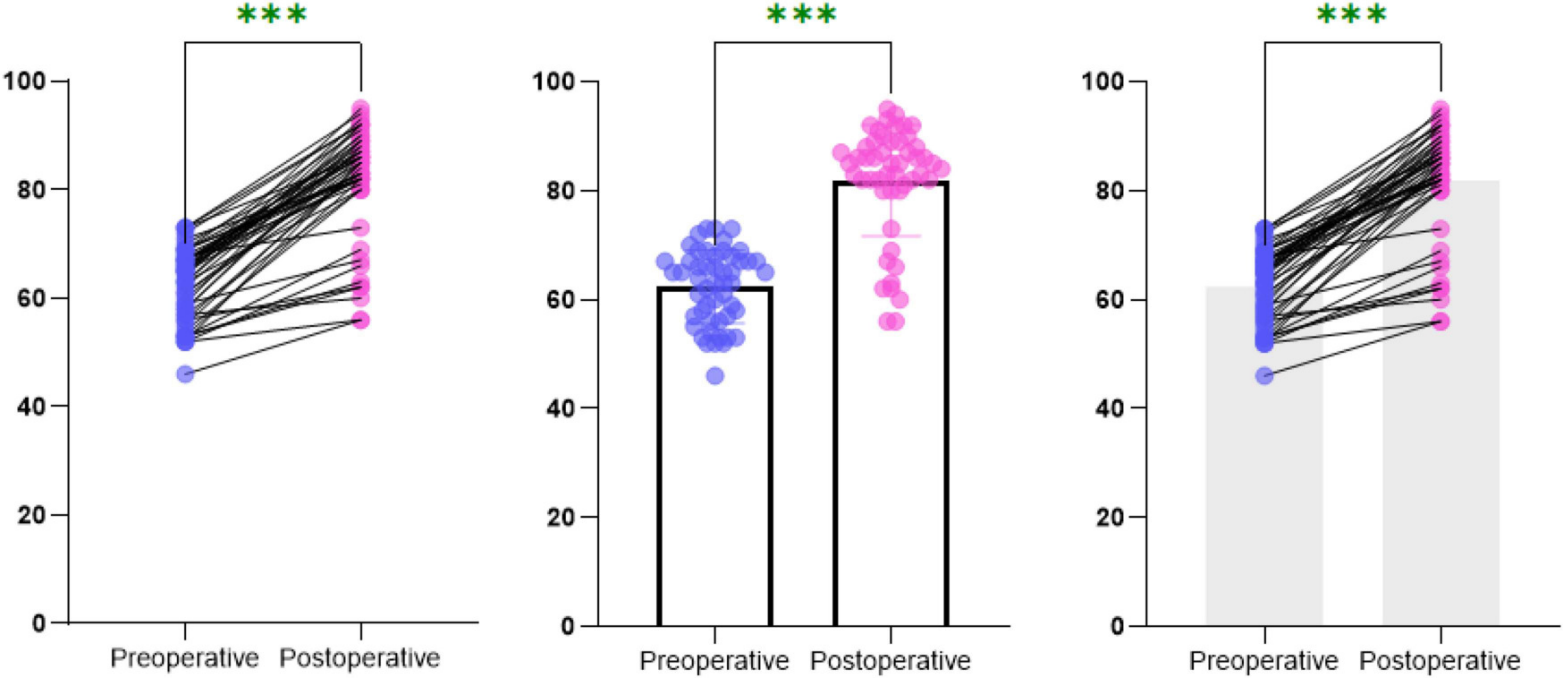
Comparison of Harris scores before surgery and at the last follow-up. The results indicated that the post-surgery Harris score was markedly elevated compared to the pre-surgery score, with the difference being statistically significant (*P* < 0.05).

**Figure 5:**
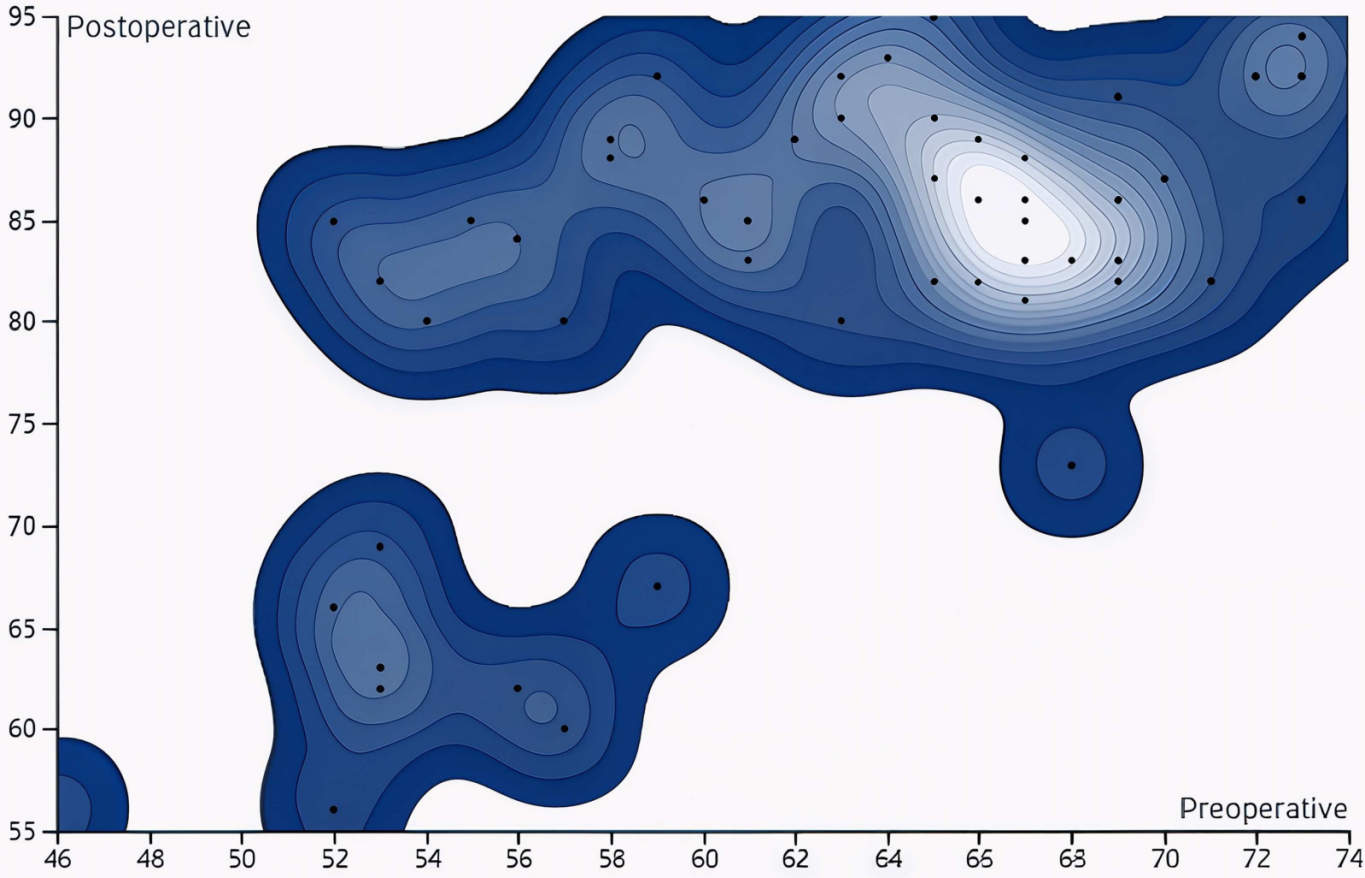
Contour distribution map of Harris scores before and after surgery at the last follow-up. The results showed that the postoperative Harris scores were mainly concentrated in the 80–90 score range, indicating that most patients had good functional recovery after the surgery.

Fifty patients (51 hips) were followed for 6–37 months (mean, 15.1 months). The preoperative Harris score was 62.39 ± 6.73 (median = 64.00, IQR = 10.00), while the final postoperative Harris score was 81.88 ± 10.20 (median = 85.00, IQR = 9.00) (Figures 4, 5). The Shapiro–Wilk test indicated that the data met the assumption of normality (*W* = 0.974, *P* = 0.310). A paired *t*-test demonstrated a statistically significant improvement (*t* = 17.58, *P* < 0.001).

Clinical success was defined as a final Harris score ≥ 80 points, no significant radiographic progression, and no conversion to THA. Based on these criteria, 41 hips (80.39%) achieved clinical success, while 10 hips (19.61%) were considered clinical failures. The reasons for failure included: progression of femoral head collapse exceeding 2 mm in 6 hips, further narrowing of the joint space in 3 hips, and insufficient functional improvement (Harris score < 70 points) despite radiographic stability in 1 hip. Radiographic success was defined as no progression of necrotic collapse or joint space narrowing, with maintenance of a continuous Shenton's line on radiographs. According to these criteria, 36 hips (70.59%) were rated as radiographic successes. Radiographs showed good integration of the transplanted iliac bone grafts with the host bone, without significant displacement or resorption. Signs of bone reconstruction began to appear at 6–8 weeks postoperatively. In successful cases, the femoral head demonstrated gradually continuous trabeculae and homogeneous density.

### 4.2 Adverse reactions

The follow-up results showed that 7 patients (7 hips) had postoperative complications, including 5 cases of poor incision healing and 2 cases of iliac bone graft displacement. The remaining patients did not have adverse events such as hip joint infection, non-healing of the incision, or deep vein thrombosis of the lower extremities. Notably, 5 patients with poor incision healing achieved good healing after timely treatment.

### 4.3 imaging evaluation

**Figure 6:**
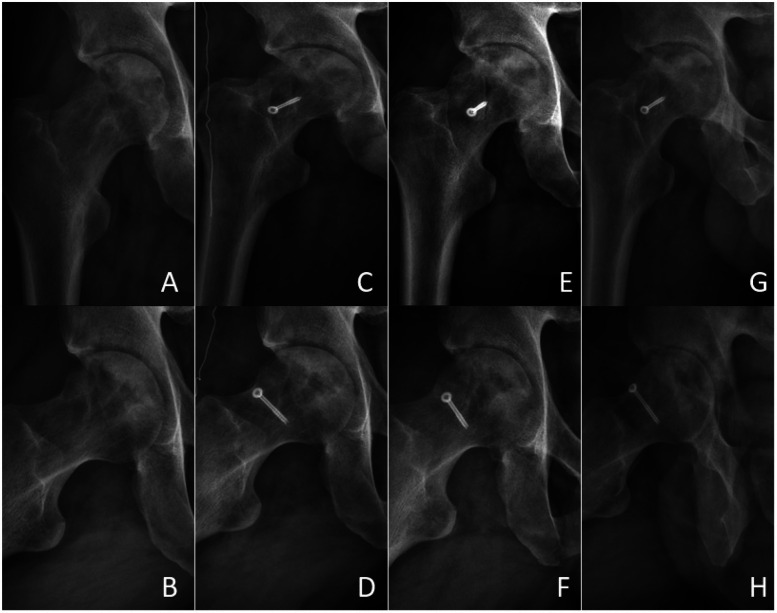
Anteroposterior and frog-leg x-ray images of the right hip joint in a typical patient. Preoperative anteroposterior and frog-leg films **(A,B)**; 1 day postoperative anteroposterior and frog-leg films **(C,D)**; 3 months postoperative anteroposterior and frog-leg films **(E,F)**; 6 months postoperative anteroposterior and frog-leg films **(G,H)**.

**Figure 7:**
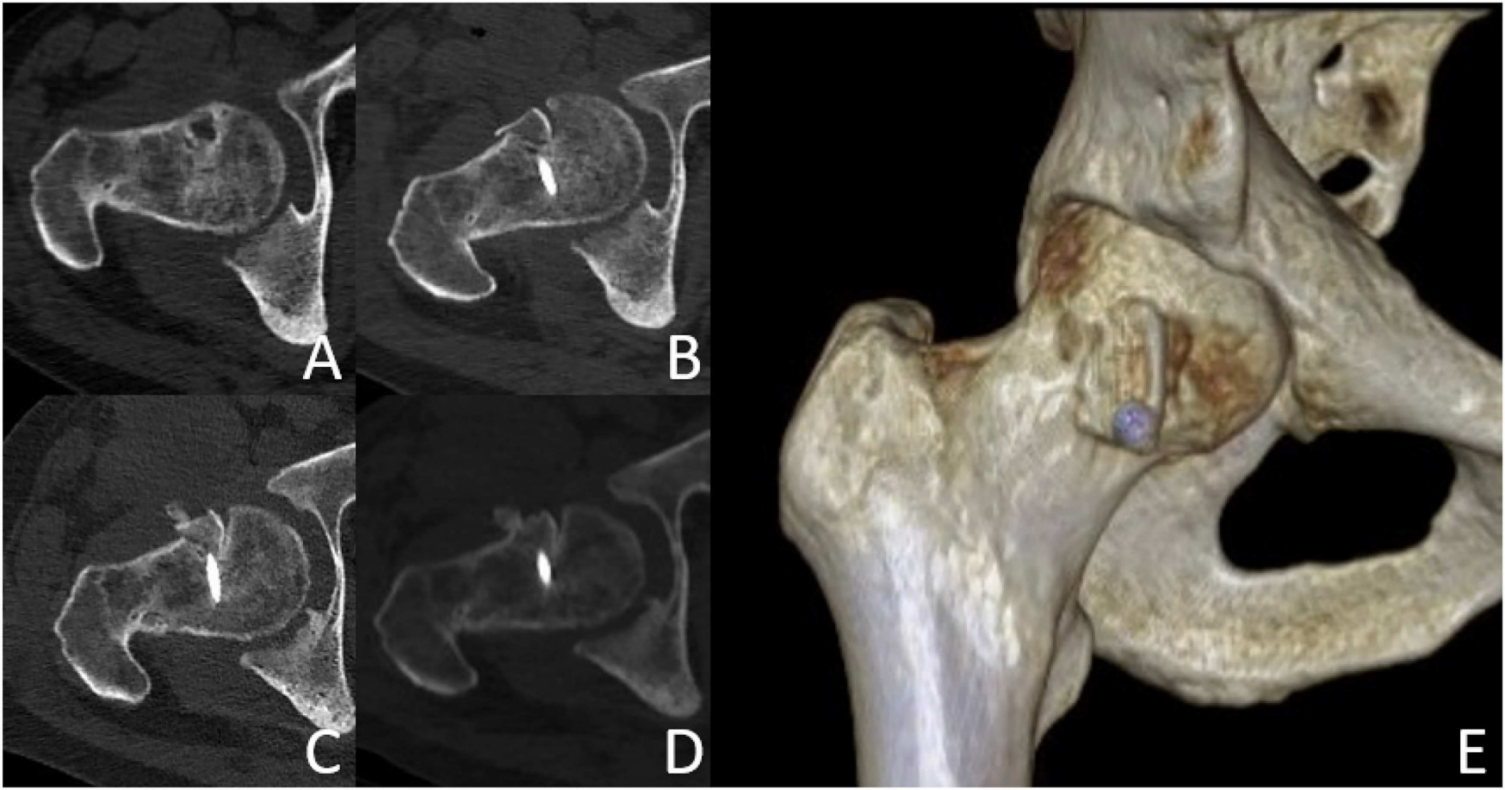
Follow-up CT and 3D reconstruction images. Preoperative CT cross-section **(A)**; CT cross-section 1 day after surgery **(B)**; CT cross-section 3 months after surgery **(C)**; CT cross-section 6 months after surgery **(D)**; 3D reconstruction image **(E)**. CT images showed sufficient filling of the bone graft area, stable shape of the femoral head, and no further collapse.

**Figure 8:**
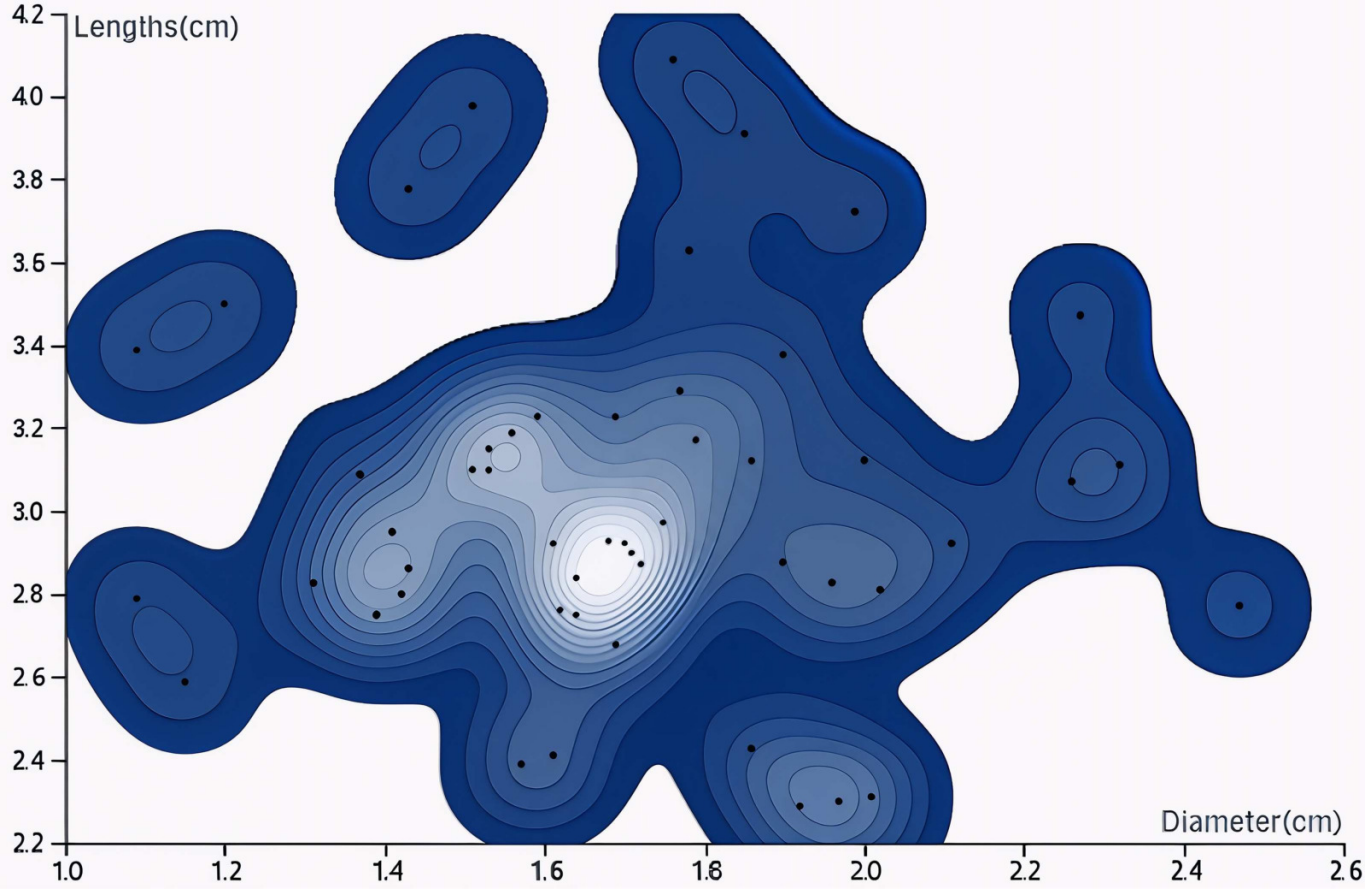
Contour distribution map of the maximum length and diameter of the implanted iliac bone graft based on CT measurement. The results showed that the length of the iliac bone graft in this study was mainly concentrated in the range of 2.5–4 cm, and the diameter was mainly concentrated in the range of 1.5–2 cm.

**Figure 9:**
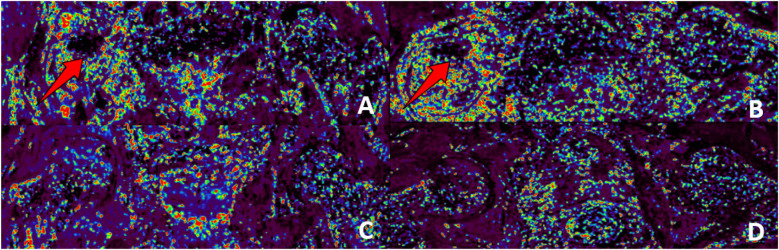
Preoperative and 6-month postoperative DCE-MRI images of the right femoral head. **(A,B)** Preoperative coronal and axial scans reveal substantial aberrant hyperperfusion in the right femoral head; **(C,D)** Postoperative coronal and axial scans showed a significant reduction in the hyperperfusion phenomena of the right femoral head, but blood perfusion in the collapsed area (shown by the arrow) has increased, suggesting partial restoration of vascular function in that region. Furthermore, the postoperative scans indicate a distinct boundary of the femoral head and a reduction in the edema of the surrounding tissues. The aforementioned results indicate that the aberrant hyperperfusion of the right femoral head is partially rectified, and the blood supply is restored post-surgery.

**Figure 10:**
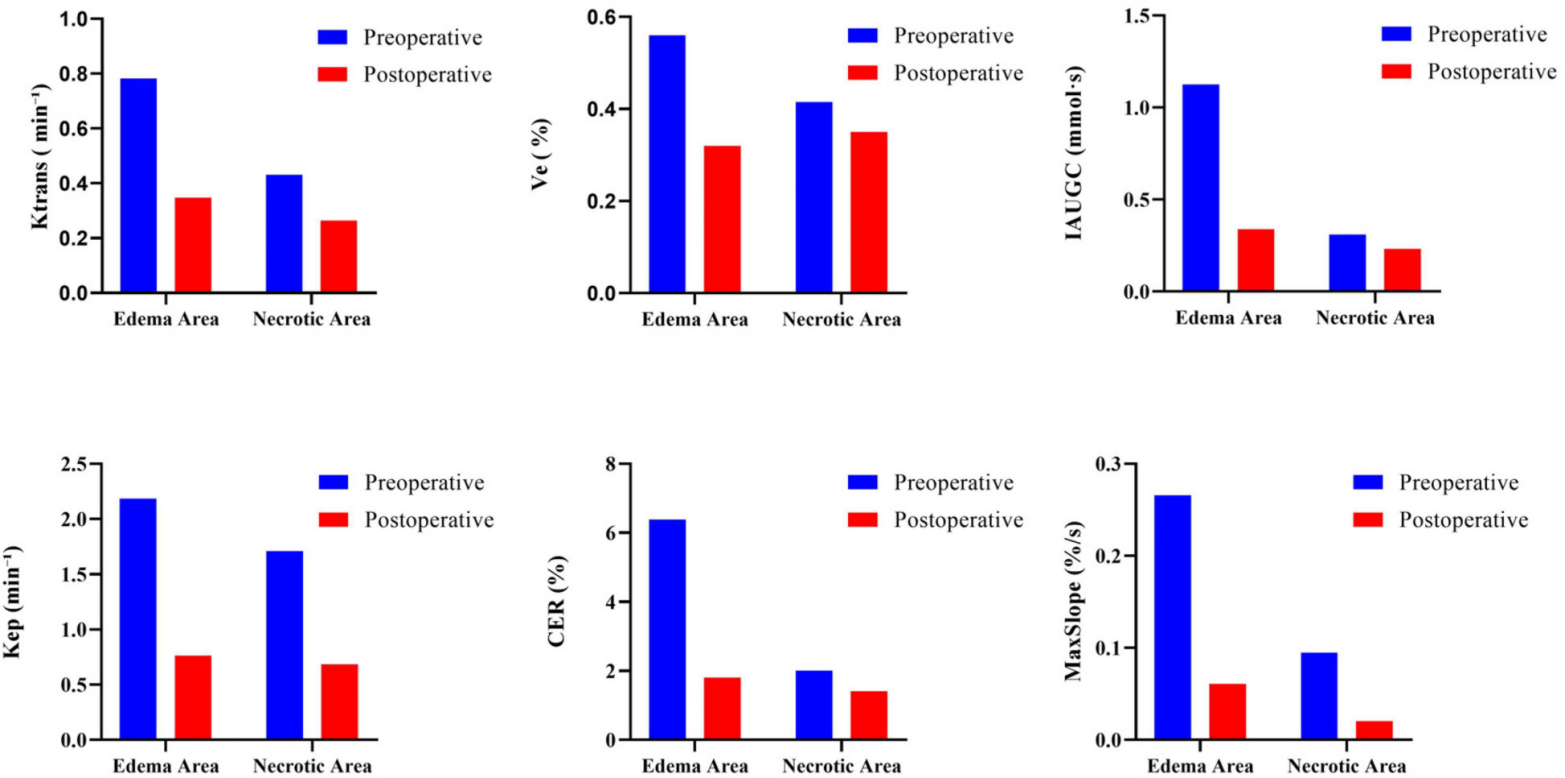
Comparison of hemodynamic parameters of the right femoral head necrosis area and edema area in typical patient before and after surgery based on DCE-MRI. Quantitative parameters include IAUGC (initial area under the gadolinium concentration–time curve, mmol·s), CER (contrast enhancement ratio, %), MaxSlope (maximum slope of the enhancement curve, %/s), Ktrans (volume transfer constant, min⁻^1^), Kep (rate constant, min⁻^1^), and Ve (extravascular extracellular volume fraction, %). The findings indicate that the parameters of IAUGC, CER, MaxSlope, Ktrans, Kep, and Ve in the necrosis area and edema area post-surgery are markedly reduced compared to pre-surgery levels, implying a potential correction of the aberrant hyperperfusion status of the right femoral head.

**Figure 11:**
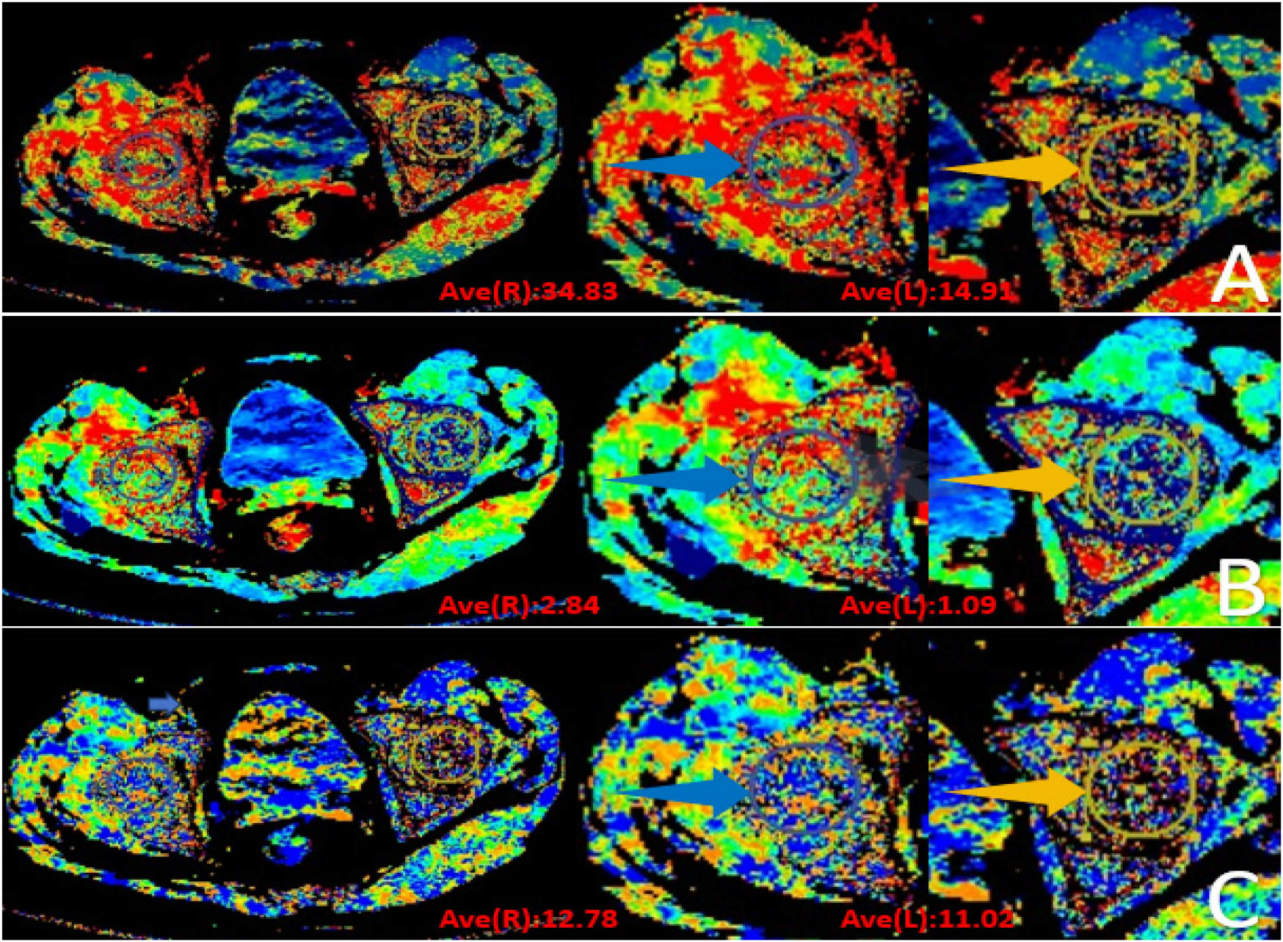
Postoperative CT perfusion (CTP) results of bilateral hip joints. **(A)** Blood flow (BF, mL/100 g/min); **(B)** Blood volume (BV, mL/100 g); **(C)** Mean transit time (MTT, s). Right femoral head indicated by blue arrow; left necrotic femoral head indicated by yellow arrow.

**Figure 12:**
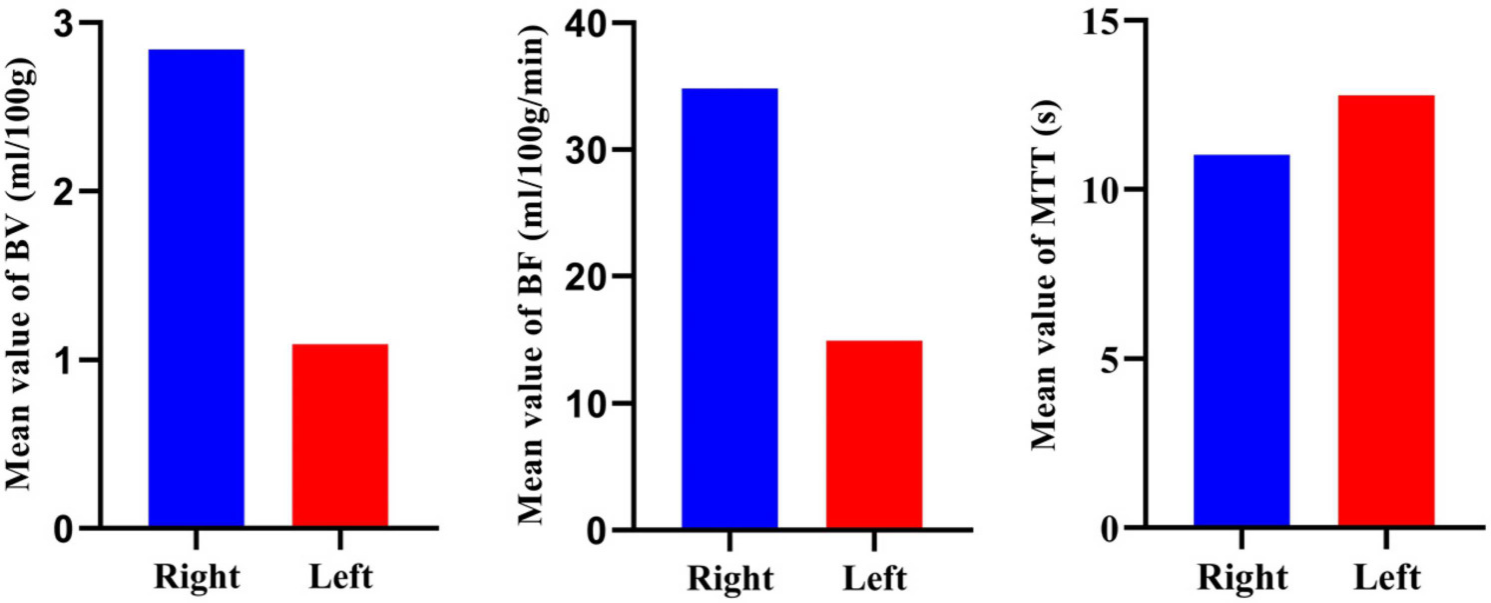
Comparison of hemodynamic parameters of bilateral femoral heads in postoperative patients. Quantitative parameters include BF (mL/100 g/min), BV (mL/100 g), and MTT (s). The results show that the average values of blood flow and blood volume of the right femoral head are higher than those of the left necrotic femoral head, while the average value of mean transit time is lower than that of the left, suggesting that the microcirculation state of the right femoral head is better than that of the left.

Typical Case: The patient, Li XX, male, a 44-year-old male, was hospitalised for “right hip joint pain with restricted mobility for over 6 months.” The physical examination revealed a markedly restricted range of motion in the right hip joint, a positive 4-character test, and a positive Thomas sign. Imaging examinations (x-ray and CT) indicated bilateral femoral head necrosis, with the lesion on the right side being markedly more severe than that on the left. See Figures 6–12.

#### 4.3.1 X-ray

See Figure 6.

#### 4.3.2 CT and 3D reconstruction

See Figures 7, 8.

#### 4.3.3 DCE-MRI

See Figures 9, 10.

#### 4.3.4 CTP

See Figures 11, 12.

### 4.4 Results of multivariate logistic regression analysis

To identify the independent factors affecting the efficacy of PVIBGT surgery, this study used multivariate Logistic regression analysis. The Block 1 model included the types of etiology and ARCO stage. The results showed that neither the type of etiology (*P* = 0.825) nor the ARCO stage (*P* = 0.620) had a significant impact on the surgical outcome. In the traumatic ONFH group, due to the small number of cases (3 cases) and consistent outcomes, a complete separation phenomenon occurred [standard error (S.E.) > 20,000], and this result had no explanatory significance. The Block 2 model included gender, age, disease duration, and the length and maximum diameter of the iliac bone graft. The results showed that neither the length of the iliac bone graft (*B* = −0.096, *P* = 0.910, OR = 0.909, 95% CI: 0.172–4.799) nor the maximum diameter of the bone graft (*B* = −0.447, *P* = 0.758, OR = 0.639, 95% CI: 0.037–11.005) was significantly correlated with the surgical success rate. Gender (*P* = 0.196), age (*P* = 0.376), and disease duration (*P* = 0.758) also showed no significant influence. The Hosmer–Lemeshow test showed *P* > 0.05, indicating a good fit of the model. In conclusion, this study did not find independent predictive factors that were significantly associated with the PVIBGT surgical outcome. See Table 2 for details.

**Table 2:** Multivariate logistic regression analysis of factors influencing surgical success after PVIBGT.

Variable	*B*	*P*	OR	95% CI for OR	Model block
Etiology		0.825			Block 1
Steroid vs. idiopathic	0.665	0.429	1.944	0.374–10.108	
Alcoholic vs. idiopathic	0.745	0.439	2.107	0.320–13.888	
Traumatic^a^	20.108	0.999	5.41 × 10⁸	—	
ARCO stage		0.620			Block 1
IIIA vs. II	−0.522	0.607	0.593	0.081–4.328	
IIIB vs. II	−0.914	0.331	0.401	0.063–2.534	
Sex (M vs. F)	1.451	0.196	4.267	0.473–38.472	Block 2
Age (years)	0.053	0.376	1.055	0.938–1.186	Block 2
Disease duration (months)	0.018	0.758	1.018	0.909–1.140	Block 2
Graft length (cm)	−0.096	0.910	0.909	0.172–4.799	Block 2
Graft maximal diameter (cm)	−0.447	0.758	0.639	0.037–11.005	Block 2
Constant	0.425	0.902	1.529	—	—

Idiopathic ONFH was used as the reference group. Hosmer–Lemeshow test *P* > 0.05 indicating good fit.

a The traumatic ONFH subgroup (*n* = 3) showed complete separation (S.E. > 20,000) and was excluded from significance interpretation.

## 5 Discussion

### 5.1 Advantages of PVIBGT in the treatment of ONFH

PVIBGT for reconstructing the blood supply of the femoral head is a reliable surgical approach for treating ONFH in ARCO stage II-IIIB (14, 29, 30). This study believes that PVIBGT in the treatment of ONFH has the following advantages: (1) Excellent biological repair: The transplanted iliac bone graft carries its own blood supply, directly delivering active cells and nutrients to the necrotic area, promoting “live bone regeneration”, simultaneously reconstructing the vascular supply of the femoral head, inhibiting inflammation and osteocyte apoptosis, and blocking the progression of the disease. (2) Stable mechanical support: The iliac bone has a thick cortical layer and abundant cancellous bone, providing support for the subchondral bone. Combined with the impaction bone grafting technique, the stability of the necrotic area can be further strengthened, significantly delaying the collapse of the femoral head. (3) Minimally invasive operation and rapid recovery: The operation is completed through the anterolateral approach, without joint dislocation, and the integrity of the joint capsule is preserved. Weight-bearing can be gradually started 3 months after the operation, especially suitable for young patients with high activity requirements. (4) Wide indications and long-lasting efficacy: It is suitable for patients in ARCO stage I-III (without collapse or mild collapse), and the 10-year hip-preserving success rate for young patients (less than 50 years old) can reach 80%–90%. (5) Low risk of complications: The main risk is vascular pedicle injury, and the incidences of infection, thrombosis, etc. are significantly lower than those of surgical methods requiring dislocation, with overall high safety.

In this study, the Harris score of the hip joint of patients at the last follow-up after the operation increased, indicating the excellent clinical efficacy of PVIBGT. This surgical technique integrates the benefits of biological and mechanical repair, addressing both the mechanical support of the necrotic region and the restoration of blood supply, while being minimally invasive and associated with low risks.

To further illustrate the clinical advantages of PVIBGT, its outcomes were compared with those reported for other hip-preserving procedures in previous studies (Table 3). Overall, the clinical success rate of our PVIBGT technique (80.4%) is comparable to or higher than most previously reported procedures.

**Table 3:** Summary of representative clinical studies on hip-preserving surgical techniques for osteonecrosis of the femoral head.

First author (Year)	Surgical technique (short)	No. of hips	Mean follow-up (months)	Clinical success/hip survival (%)
Xie (31) (2019)	Pedicled vascularized iliac bone graft (PVIBGT)	1,006	180	88.9%
Yoo (32) (2008)	Free vascularized fibular graft (FVFG)	124	167	89.5%
Baydar (33) (2022)	Free vascularized fibula graft (FVFG)	54	66	72.2%
Lau (34) (2021)	Vascularized iliac bone graft (VIBG)	50	146	56.0%
Liu (35) (2014)	Porous tantalum rod implantation (PTR)	138	38.5	69.0%
Zhang (36) (2021)	Porous tantalum rod implantation (PTR)	52	85.7	53.8%
Chen (37) (2020)	Core decompression + non-vascularized allogeneic fibular graft (CD + NVAFG)	44	89	81.8%
Sallam (38) (2017)	Core decompression + autogenous bone graft (CD + ABG)	71	94	72.7%
This manuscript	Pedicled vascularized iliac bone graft transfer (PVIBGT)	51	15.1	80.4%

ABG, autogenous bone graft; CD, core decompression; FVFG, free vascularized fibular graft; NVAFG, non-vascularized allogeneic fibular graft; PTR, porous tantalum rod implantation; PVIBGT, pedicled vascularized iliac bone graft transfer; VIBG, vascularized iliac bone graft; THA, total hip arthroplasty. Data were summarized from published clinical studies reporting follow-up duration and hip-preservation or clinical-success rate.

### 5.2 Advantages of DCE-MRI and CTP in evaluating femoral head blood supply

Dynamic enhanced MRI (DCE-MRI) is a non-invasive method employed to evaluate the perfusion characteristics of a lesion, primarily focusing on the exchange dynamics between blood within capillaries and the extravascular extracellular space (39). This technique reflects microvascular distribution and capillary blood flow perfusion, assessing the viability and function of local tissues while specifically evaluating the perfusion status of tissues and organs. CTP is a CT-based method that evaluates the blood supply status of the femoral head concerning blood flow function. CTP quantifies local blood flow (BF), blood volume (BV), and mean time to transit (MTT), which are directly the integration of DCE-MRI and CTP addresses the limitations inherent in a single modality, thereby enhancing the timing of hip-preserving surgery and facilitating the assessment of treatment efficacy.

In ONFH, venous stasis and return blood blockage occur in the early stage, arterial ischemia in the middle stage, and arterial occlusion in the late stage. When the femoral head is in the “peri-collapse stage”, x-rays and CT cannot fully reflect the real situation, and a comprehensive evaluation combined with the blood supply of the femoral head is still required. After the diagnosis of osteonecrosis, patients who are often recommended to undergo hip joint preservation surgery are advised to undergo digital subtraction angiography (DSA) to provide a basis for formulating the surgical plan. However, DSA has problems such as greater trauma, inability to capture microcirculation disorders, and the need for hospitalization, and DSA has low sensitivity to early ONFH. In contrast, DCE-MRI and CTP can complete follow-up examinations in outpatient clinics, only require intravenous injection, have small trauma, low risk, no need for hospitalization, and are more sensitive to microcirculation changes, thus having obvious advantages in evaluating the blood supply of the femoral head (40–43).

This study compares DCE-MRI images obtained before and after PVIBGT, revealing that the abnormal high perfusion of the necrotic femoral head was partially corrected, with the blood supply in the necrotic area gradually normalising post-operation. CTP examination showed that the perfusion distribution was uniform and the hemodynamic performance was good in the reconstructed femoral head on the surgical side; while in the non—operated necrotic femoral head on the contralateral side, perfusion was uneven and blood flow was low. These results suggest that PVIBGT may contribute to the formation of a more uniform local blood flow distribution within the necrotic femoral head. Since this study did not perform preoperative CTP examination and lacked histological or molecular verification, it was not possible to directly confirm the mechanism of action of PVIBGT in angiogenesis or osteocyte protection. Therefore, the conclusions of this study were mainly based on radiological and clinical observations. The results showed that PVIBGT could achieve stable structural repair and functional recovery in some ONFH patients, and this effect may be related to the reconstruction of a stable blood supply within the femoral head.

### 5.3 Analysis of the causes of hip preservation failure

Currently, the removal of necrotic bone tissue to the maximum extent and avoiding iatrogenic injury, as well as how to correctly implant the iliac bone graft into the necrotic area after removing the dead bone have become the key to the treatment of ONFH. In this study, a total of 10 cases (19.61%) experienced disease progression during the follow-up period. Among them, 6 hips showed progression of femoral head collapse exceeding 2 mm, 3 hips exhibited further narrowing of the joint space accompanied by persistent pain, and 1 hip had insufficient functional improvement (Harris score < 70) despite radiographic stability. Causes of failure included continued postoperative steroid use (2 cases), improper early postoperative weight-bearing (2 cases), incomplete filling of the necrotic cavity (2 cases), and poor compliance with systematic rehabilitation (4 cases). All failed cases ultimately underwent total hip arthroplasty. Radiographically, these cases often presented with heterogeneous density of the femoral head, poor trabecular bone reconstruction, bone resorption at the graft—host bone junction, or bone graft displacement. It is worth noting that some studies have shown that the success rate of hip preservation in this surgical method is low in specific situations. For example, Chen et al. (44) treated 32 patients (33 hips) with collapsed femoral heads at ARCO stage IIIa and IIIb with this surgical method. The average follow-up was 69 months after the operation, and the hip joint survival rate was only 24% (8/33), and all were patients at preoperative ARCO stage IIIa. Therefore, they believed that this surgical method was not suitable for patients with large collapsed femoral heads. Meyers (45) first reported the use of pedicled iliac bone graft transplantation to treat 23 ONFH patients. Among them, 8 patients at Ficat stage I or II achieved satisfactory results, while only 1/3 of the patients at Ficat stage III or IV had satisfactory results. Therefore, in order to avoid hip preservation failure as much as possible, this study summarizes the following possible causes of hip preservation failure (46–49): (1) The stage of necrosis is too late (ARCO IIIC–IV stage): The femoral head has undergone obvious collapse or secondary osteoarthritis (ARCO IV stage), at this time, the subchondral bone structure damage is irreversible, and it is difficult to restore mechanical stability with simple bone graft transplantation. (2) Vascular pedicle injury or failure of blood supply reconstruction: Inadequate free vascular pedicle during the operation, thrombosis at the anastomosis site or vascular compression after the operation, resulting in interruption of the blood supply of the bone graft and inability of the transplanted bone to survive. (3) The bone graft is not firmly fixed or the mechanical support is insufficient: In the early stage of this group, it was simply embedded and easily not firmly fixed, resulting in mechanical support failure and inability to resist the weight-bearing pressure of the femoral head. Later, titanium nail fixation was changed, and the effect was better. In addition, if the bone graft does not fully fill the necrotic area, it is also easy to lead to hip preservation failure. (4) Early weight-bearing or improper rehabilitation after the operation, resulting in excessive pressure on the incompletely healed bone graft and accelerated collapse. (5) High-risk factors of patients are not controlled: The primary cause (such as long-term hormone use, alcohol abuse) is not corrected, resulting in recurrence or progression of necrosis. (6) Anatomical variations in some patients, thin ascending branches of the lateral rotary femoral artery, poor incision healing after surgery, etc. See Table 4 for details.

**Table 4:** Causes of hip preservation failure in patients undergoing PVIBGT.

Causes of failure	Underlying mechanisms
Advanced necrosis stage	Femoral head collapse >2 mm or osteoarthritis (ARCO stage IIIC–IV), irreversible subchondral bone destruction
Vascular pedicle injury or blood supply failure	Inadequate vascular pedicle dissection, anastomotic thrombosis, or compression leading to bone graft ischemia
Insufficient bone graft fixation or support	Unstable initial inlay fixation; insufficient bone graft filling, leading to mechanical support failure
Premature postoperative weight-bearing	Early weight-bearing or improper rehabilitation causing mechanical damage to the unhealed bone graft
Uncontrolled high-risk factors	Persistent steroid/alcohol abuse contributing to recurrent necrosis
Anatomical variations	Thin deep ALFCA, etc.
Poor wound healing	Postoperative wound infection or other complications

Limitations observed during the surgical operation: (1) It is difficult to obtain sufficient cancellous bone on the ilium of lean patients, and allogeneic bone is required for patients with a large necrotic area; (2) Iatrogenic injuries are prone to occur during the operation, such as injury to the iliac bone graft, ilium, subchondral bone, and tensor fascia lata.

Several patients experienced inadequate incision healing or fat liquefaction postoperatively, with the incisions ultimately healing with debridement. Upon analysis, the potential reasons are as follows: (1) The iliac flap transplantation utilizing the ascending branch of the lateral circumflex femoral artery employs the S-P incision technique. Excessive removal of bone grafts and cancellous bone near the anterior superior iliac spine can lead to the formation of a subcutaneous cavity, resulting in fluid accumulation that impairs incision healing; (2) The skin at the fenestration site is delicate and in proximity to the subcutaneous tissue; (3) The groin region contains numerous sweat glands, increasing the likelihood of sweat accumulation; (4) The elastic band of the patient's shorts is in close contact with the incision, potentially causing friction that hinders healing.

### 5.4 Clinical significance of the results of multivariate analysis

The results of the multivariate analysis in this study indicated that the etiology type, ARCO stage, gender, age, disease duration, and the size of the iliac bone graft (length and maximum diameter) were not independent predictive factors for postoperative success. This finding was consistent with some existing studies. Lei et al. (15) pointed out that under standardized surgical procedures, the morphological size of the iliac bone graft had limited influence on short—term efficacy, and the quality of postoperative blood circulation reconstruction and bone integration might be more crucial (50–52). Most existing literature recommends an iliac bone graft length of 4–5 cm and a width of 2–3 cm. This range can provide sufficient mechanical support while ensuring blood supply safety (44, 53). There were certain differences between the measured values of the bone graft in this study and the recommended range in the literature, which might be related to the measurement method: in this study, the length and maximum diameter of the bone graft were measured based on CT cross-sections, which might have a systematic error compared with the actual anatomical dimensions during surgery. In addition, an overly large iliac bone graft size might increase the tension of the vascular pedicle, while an overly small size might result in insufficient support. Therefore, the appropriate size should be individually designed based on the range of the necrotic area and the individual acetabular structure (16, 54–56).

The fact that this study did not find significant predictive factors might be related to the limited sample size, high case heterogeneity, and the small number of cases in some subgroups (such as traumatic ONFH). In the future, the sample size can be expanded through multi-center cooperation, and combined with three-dimensional reconstruction technology, to further explore the quantitative relationship between the geometric parameters of the iliac bone graft and surgical efficacy.

### 5.5 Limitations of this study and next steps

This study has several limitations. First, the small sample size (51 cases) may limit the representativeness of the findings. Second, as a retrospective study without a control group, it is subject to selection bias and has limited ability to establish causality; therefore, caution should be exercised when attributing the observed short-term outcomes or imaging changes to PVIBGT. Third, CTP examinations were only performed postoperatively, and the lack of preoperative data prevents accurate assessment of perfusion improvement. Due to the retrospective nature of the study, preoperative CTP data were unavailable, and for ethical considerations regarding radiation exposure, patients were not required to undergo additional preoperative scans for research purposes. Fourth, the short follow-up period only reflects short-term efficacy. Fifth, variations in preoperative disease duration and prior treatments among patients may have influenced postoperative outcomes. Sixth, current DCE-MRI and CTP evaluations of femoral head blood supply lack standardized quantitative criteria, and measurements are susceptible to variations in scanning parameters, patient positioning, and timing.

Future studies should adopt a prospective, multi-center, large-sample design and establish standardized imaging protocols to minimize bias and errors. Pre- and postoperative perfusion imaging should be included to more accurately assess hemodynamic changes following PVIBGT. In addition, control groups receiving conservative treatment or alternative surgical approaches should be incorporated, follow-up periods extended, and unified quantitative imaging standards established to further validate the long-term efficacy of PVIBGT.

## 6 Conclusion

Our findings indicate that PVIBGT can improve hip joint function in patients with ONFH and delay the progression of femoral head collapse. Postoperatively, patients showed significant improvement in hip function, with markedly increased Harris scores and a clinical success rate of 80.39%. Imaging follow-up demonstrated stable integration of the transplanted iliac bone graft with the femoral head, uniform perfusion distribution in the reconstructed area, and favorable postoperative structural remodeling. Multivariate logistic regression analysis revealed that etiology type, ARCO stage, sex, age, disease duration, and bone graft size were not independent predictors of postoperative success. This suggests that, beyond basic demographic and morphological indicators, the quality of postoperative revascularization and bone integration may play a more critical role in long-term outcomes.

Furthermore, this study highlights the significant value of DCE-MRI and CTP in evaluating femoral head blood supply, providing an objective basis for efficacy monitoring. While CTP is widely used in cerebral infarction research, the interpretation of its parameters in assessing femoral head perfusion requires further investigation. Our research group is currently in the preliminary stages of studying DCE-MRI and CTP, and preoperative quantitative analysis was not performed in some cases. Statistical analysis of the collected data is still ongoing, and further in-depth studies are warranted. Future research should adopt a prospective, multi-center, large-sample design, establish standardized imaging protocols, and incorporate pre- and postoperative perfusion imaging to further validate the role of PVIBGT in revascularization and develop quantifiable imaging indicators predictive of therapeutic outcomes.

## Data Availability

The original contributions presented in the study are included in the article/Supplementary Material, further inquiries can be directed to the corresponding authors.
